# Retraction: The Association Between Janus Kinase 2 and Factor V Leiden Mutations and Thrombotic Complications in Patients With Myeloproliferative Disorders: A Study From Saudi Arabia

**DOI:** 10.7759/cureus.r232

**Published:** 2026-07-02

**Authors:** Wafaa S Sayed, Aws Al-Bayati, Lina G Elzubair, Sherif Mohamed, Mueed Alharthi

**Affiliations:** 1 Clinical Pathology, Aswan University Hospital, Aswan, EGY; 2 Clinical Pathology, Khamis Mushait General Hospital, Khamis Mushait, SAU; 3 Clinical Hematology, Armed Forces Hospital - Southern Region, Khamis Mushait, SAU; 4 Haematopathology, Armed Forces Hospital - Southern Region, Khamis Mushait, SAU; 5 Chest Diseases and Tuberculosis, Faculty of Medicine, Assiut University, Assiut, EGY; 6 Internal Medicine, Prince Sultan Bin Abdulaziz Humanitarian City, Riyadh, SAU; 7 Clinical Hematology, Khamis Mushait General Hospital, Khamis Mushait, SAU

This article has been retracted by the Editor-in-Chief due to plagiarism and gift authorship. Specifically, this article plagiarized content from the following Master's thesis: "The Association between Janus Kinase 2 (JAK 2) and Factor 5 Leiden (FVL) Mutation on the Thrombotic among Patients with Myeloproliferative Disorder" by Tofaha Sulaiman Abas Al-Twaissi (Al-Ahliyya Amman University, Jordan. August, 2023).

The authors agree with the decision to retract.

